# The Malleability of Gold

**Published:** 1891-02

**Authors:** 


THE MALLEABILITY OF GOLD.—The gold beaters of Berlin, at the Paris exposition, showed gold leaves so thin that it would require 282,000 to produce the thickness of a single inch, yet each leaf is so perfect and free from holes as to be impenetrable by the strongest electric light. If these leaves were bound in book form it would take 15,000 to fill the space of ten common bookleaves.

